# Whole Rye Consumption Improves Blood and Liver n-3 Fatty Acid Profile and Gut Microbiota Composition in Rats

**DOI:** 10.1371/journal.pone.0148118

**Published:** 2016-02-10

**Authors:** Fayçal Ounnas, Florence Privé, Patricia Salen, Nadia Gaci, William Tottey, Luca Calani, Letizia Bresciani, Noelia López-Gutiérrez, Florence Hazane-Puch, François Laporte, Jean-François Brugère, Daniele Del Rio, Christine Demeilliers, Michel de Lorgeril

**Affiliations:** 1 Laboratoire TIMC-IMAG CNRS UMR 5525, Equipe PRETA, Cœur et Nutrition, Université Joseph Fourier, Grenoble I, France; 2 Laboratory of Fundamental and Applied Bioenergetics (LBFA), Université Grenoble Alpes, Grenoble, France; 3 Inserm, U1055, Grenoble, France; 4 EA 4678 CIDAM, Clermont-Université, Université d'Auvergne, Clermont-Ferrand, France; 5 LS9 Interlab Group, Laboratory of Phytochemicals in Physiology, Department of Food Science, University of Parma, Medical School, Building C, Via Volturno 39, 43125 Parma, Italy; 6 Département de Biochimie, Pharmacologie et Toxicologie, Unité Biochimie Hormonale et Nutritionnelle, Centre Hospitalier et Universitaire de Grenoble, Grenoble, France; 7 Department of Chemistry and Physics (Analytical Chemistry Area), Research Centre for Agricultural and Food Biotechnology (BITAL), Agrifood Campus of International Excellence, ceiA3, University of Almería, Carretera de Sacramento s/n, E-04120 Almería, Spain; Wageningen University, NETHERLANDS

## Abstract

**Background:**

Whole rye (WR) consumption seems to be associated with beneficial health effects. Although rye fiber and polyphenols are thought to be bioactive, the mechanisms behind the health effects of WR have yet to be fully identified. This study in rats was designed to investigate whether WR can influence the metabolism of n-3 and n-6 long-chain fatty acids (LCFA) and gut microbiota composition.

**Methods:**

For 12 weeks, rats were fed a diet containing either 50% WR or 50% refined rye (RR). The WR diet provided more fiber (+21%) and polyphenols (+29%) than the RR diet. Fat intake was the same in both diets and particularly involved similar amounts of *essential* (18-carbon) n-3 and n-6 LCFAs.

**Results:**

The WR diet significantly increased the 24-hour urinary excretion of polyphenol metabolites–including enterolactone–compared with the RR diet. The WR rats had significantly more n-3 LCFA–in particular, eicosapentanoic (EPA) and docosahexanoic (DHA) acids–in their plasma and liver. Compared with the RR diet, the WR diet brought significant changes in gut microbiota composition, with increased diversity in the feces (Shannon and Simpson indices), decreased *Firmicutes/Bacteroidetes* ratio and decreased proportions of uncultured Clostridiales cluster IA and *Clostridium* cluster IV in the feces. In contrast, no difference was found between groups with regards to cecum microbiota. The WR rats had lower concentrations of total short-chain fatty acids (SCFA) in cecum and feces (p<0.05). Finally, acetate was lower (p<0.001) in the cecum of WR rats while butyrate was lower (p<0.05) in the feces of WR rats.

**Interpretation:**

This study shows for the first time that WR consumption results in major biological modifications–increased plasma and liver n-3 EPA and DHA levels and improved gut microbiota profile, notably with increased diversity–known to provide health benefits. Unexpectedly, WR decreased SCFA levels in both cecum and feces. More studies are needed to understand the interactions between whole rye (fiber and polyphenols) and gut microbiota and also the mechanisms of action responsible for stimulating n-3 fatty acid metabolism.

## Introduction

The consumption of whole grain cereals seems to be linked with a reduced risk of chronic disease [1]. However, the biological mechanisms responsible for these effects have yet to be identified [2,3]. Rye is a cereal commonly consumed in wholegrain form by European populations [4,5]. Whole rye (WR) is rich in several potentially bioactive compounds found in the bran, the outer part of the grain [5–7]. For instance, the polyphenols lignans–which are converted by mammalian gut bacteria into enterodiol and enterolactone [6–8]–are antioxidant and express hormonal activity thought to reduce the risk of hormone-mediated diseases [8]. They may also interact with n-3 long-chain fatty acids (LCFA) metabolism, resulting in potential health benefits [9]. Whereas the endogenous n-3 LCFA metabolism is partly regulated by sex hormones [10–12], it is unknown whether rye polyphenols–which have hormonal activity [6–8]–can affect n-3 metabolism. Indeed it has been reported that cereal polyphenols such as anthocyanins (from black corn) and phenolic acids (from wheat aleurone) increase plasma n-3 LCFA–eicosapentanoic (EPA) and docosahexanoic (DHA) acid–concentrations in rats [13,14]. Like most cereals, WR is an important source of fiber, especially arabinoxylans [15], which are substrate for bacterial fermentation in the gastrointestinal tract [16]. The gut microbiota consists of several hundred species of bacteria potentially important for human health [17]. Whether rye fiber increase fermentation in the gastrointestinal tract and selectively stimulates some groups of bacteria has yet to be fully investigated [18]. Rye fiber may increase the bacteria-induced generation of short-chain fatty acids (SCFA), which are also considered beneficial for health [19–21]. The specific composition and activity of gut microbiota (at phyla, genus or species level) are known to affect host metabolism and promote either health or alternatively various diseases, including diabetes and obesity [22,23]. In fact, the effects of WR consumption on these pathological states, either through its effect on gut microbiota or otherwise, are not fully understood. Considering the current shortage of information on how WR provides health benefits [24], this work in rats was primarily designed to investigate whether WR consumption, as compared with refined rye (RR), can affect n-3 and n-6 LCFA metabolism and gut microbiota composition.

## Materials and Methods

### Chemicals

Sodium hydroxide, citric acid, *trans*-ferulic acid, *trans*-isoferulic acid, sinapic acid, caffeic acid, 4-hydroxybenzoic acid, 3’-coumaric acid, 4’-coumaric acid, vanillic acid (3-methoxy-4-hydroxybenzoic acid), protocatechuic acid (3,4-dihydroxybenzoic acid), 3’,4’-dihydroxyphenylacetic acid, 3-(3’-hydroxyphenyl)propionic acid, 3-(4’-hydroxyphenyl)propionic acid, 3-(3’,4’-dihydroxyphenyl)propionic acid (aka dihydrocaffeic acid), 3-(3’,5’-dihydroxyphenyl)propionic acid, 3-(3’-methoxy-4’-hydroxyphenyl)propionic acid (aka dihydroferulic acid), and hippuric acid were purchased from Sigma-Aldrich (St. Louis, MO, USA) as were secoisolariciresinol, enterolactone and enterodiol. Homovanillic acid (3’-methoxy-4’-hydroxyphenylacetic acid) was purchased from Extrasynthese (Genay Cedex, France). Ferulic acid-4’-O-sulphate disodium salt, isoferulic acid-3’-O-β-D-glucuronide, and dihydrocaffeic acid-3’-O-sulphate sodium salt were purchased from Toronto Research Chemical, (Toronto, Canada). Daidzein was purchased in part from AASC. Ltd (Southampton, UK) and in part provided by Prof. Alan Crozier (Department of Nutrition, University of California, Davis, CA, USA).

### Experimental diets

Two experimental diets were prepared in this study. The first was obtained by mixing the standard diet flour (A03, SAFE Diets, France) with 50% refined rye (RR). The second was prepared by mixing the same standard diet A03 flour with 50% whole rye (WR). The amount of nutrients provided by the experimental diets was adjusted to the recommendations of the American Institute of Nutrition Rodent Diets-93 [25].

### Animals and experimental design

The rats were cared for in accordance with European Council Directive 86/609/EEC on the care and use of laboratory animals (OJ L 358). Protocols were carried out under license from the French Ministry of Agriculture (Permit Number: n° A380727) and approved by the Committee on the Ethics of Animal Experiments of the University of Grenoble (Permit Number: n^o^ 113_ LBFA-FO-01). All efforts were made to minimize suffering.

Twenty-four male Wistar rats (Charles River Laboratories, l’Arbresle, France, baseline body weight 75–100 g) were fed a standard diet (A04, SAFE Diets, France). They were housed in individual cages under conditions of constant temperature and humidity and a standard light-dark cycle (12 h/12 h). Tap water and standard diet were provided *ad libitum*.

The animals were acclimatized one week before being randomly distributed into two groups (n = 12/group). The rats were then fed either the RR or the WR diet for 12 weeks. Weight and food consumption were recorded weekly. One week before they were sacrificed, the rats' urine was sampled in individual metabolic cages. At the end of the experiment, the rats' plasma was sampled and stored at -80°C and the liver was dissected and stored at -80°C. For bacterial analysis, freshly voided feces were sampled in sterile tubes at the start and end of the experiment (F0 and F12 respectively). The cecal content (Cae) was dissected and sampled in sterile tubes at the time of sacrifice. All samples were immediately frozen at -80°C until further analysis.

### Fatty acid analyses

Plasma and liver lipids were extracted in hexane/isopropanol as described above [26,27]. Plasma was selected as the main biological component involved in fatty acid distribution in the body. The liver is the main organ where the endogenous synthesis of n-3 LCFA occurs in mammals. In short, methylated fatty acids were extracted with hexane, separated, and quantified by GC using a 6850 Series gas chromatograph system (Agilent Technologies, Palo Alto, CA, USA). Methyl ester peaks were identified by comparing their retention time to those of a standard mixture. Saturated, mono-, and poly-unsaturated fatty acid levels were expressed as a percentage of total fatty acids. Total cholesterol and triglycerides were measured using standard methodology on a Synchron Clinical System LX20 (Beckma Coulter, Brea, CA, USA).

### Analysis of polyphenolic compounds from refined and whole rye diets

Polyphenolic compounds were analyzed using an Accela UHPLC 1250 equipped with a linear ion-trap mass spectrometer (LTQ XL, Thermo Fisher Scientific Inc., San Jose, CA, USA) fitted with a heated-electrospray ionization probe (H-ESI-II; Thermo Fisher Scientific Inc.). Separation was performed using a BlueOrchid-1.8 C18 column (50 × 2 mm) (Knauer, Berlin, Germany). Pure helium gas (99.9%) was used for collision-induced dissociation. The phenolic acid fraction was extracted from rye pellets and analyzed using the same method as reported above [14,28]. Other polyphenolic compounds such as lignans and flavonoids were extracted differently. In short, 150 mg of sample was defatted with 1.5 mL of hexane, vortexed for 2 minutes and centrifuged at 9200 g for 15 minutes. Then the supernatant was removed. Polyphenols were extracted from the residue using 2.5 mL of acidified MeOH:H2O (1M with HCl), 70:30 v/v. The mixture was refluxed for 1 h at 70°C in a water bath. Then, the samples were cooled and neutralized (pH 5–6) with NaOH, 2M. After that, 3 mL of ethyl acetate was added and samples were agitated briefly for 1 minute in a vortex. The samples were then centrifuged at 9200 g for 15 minutes. Later, 1.5 mL of extract was reduced to dryness in vacuo and the residue was dissolved in a mobile phase mixture (MeOH:water 0.1% and formic acid, 50:50, v/v) and filtered prior to UHPLC-MS^n^ analysis. In detail, isoflavone and lignan analysis was carried out using full scan, data-dependent MS^3^ scanning from *m/z* 100 to 1500 in the negative ion mode. The capillary T was 275°C while the source was maintained at 200°C. The sheath gas flow was 40 units, while auxiliary and sweep gases were set to 10 and 2 units, respectively. The source voltage was 4 kV. The capillary and tube lens voltages were -24 and -108 V, respectively. For UHPLC, mobile phase A was 0.1% formic acid in water and phase mobile B was acetonitrile with 0.1% formic acid. The mobile phase was pumped at a flow rate of 0.2 mL/min, with 0–2 min at 5% of B and 3–12 min at 5% to 50% of B. CID = 30 was used for MS^2^ and MS^3^ experiments. Isoflavones were quantified in daidzein equivalent in full scan mode by extracting the relative molecular ion for each compound, while lignans were quantified in the same mode but in secoisolariciresinol equivalent. Xcalibur software (Thermo Fisher Scientific Inc., San Jose, CA, USA) was used to perform data analysis.

### Analysis of urinary phenolic metabolites

Urine samples of rats were diluted with 0.1% aqueous formic acid and filtered through a 0.45 μm nylon filter prior to UHPLC-MS^n^ analysis using an Accela UHPLC 1250 equipped with linear ion-trap mass spectrometer (LTQ XL, Thermo Fisher Scientific Inc., San Jose, CA, USA) fitted with a heated-electrospray ionization probe (H-ESI-II; Thermo Fisher Scientific Inc.). A preliminary analysis was carried out using full scan, data-dependent MS^3^ scanning from *m/z* 100 to 800 to carry out an initial investigation of the main urinary metabolites. Based on information obtained, the main urinary metabolites were monitored in MS^2^ or MS^3^ mode. The MS worked in negative ionization mode, with capillary temperature equal to 275°C, while the source heater was set to 250°C. The sheath gas flow was 40 units, while auxiliary and sweep gases were set to 5 units. The source voltage was 3 kV. The capillary voltage and tube lens were -5 and -68 V, respectively. For UHPLC, mobile phase A was 0.1% formic acid in water and mobile phase B was acetonitrile containing 0.1% formic acid. Separations were performed using a Kinetex PFP column (50 × 2.1 mm) with, 2.6 μm particle size (Phenomenex, Torrance, CA, USA). The mobile phase, pumped at a flow rate of 0.2 mL/min, comprised 0–1 minutes at 2% B followed by 1–12 minutes at 2% to 35% B. All metabolites were fragmented in MS^2^ or MS^3^ using a CID of 30, with the sole exception of enterodiol and enterolactone, for which a CID of 40 in MS^2^ (for free forms) and in MS^3^ (for conjugated forms) was used. Helium gas was used for CID. Free forms of phenolic acids were quantified in MS^2^ mode, while phenolic acid conjugates were quantified in MS^3^ mode, except for enterodiol and enterolactone metabolites, which were quantified using selective ion monitoring (SIM). 3-(3’,5’-dihydroxyphenyl)propionic acid was quantified by means of the corresponding commercial standards, while 3’-coumaric acid was quantified as 4’-coumaric acid. Benzoic acid-*O*-sulphate and caffeic acid-*O*-sulphate were quantified as 3-(4’-hydroxyphenyl)propionic acid-3’-*O*-sulphate (aka dihydrocaffeic acid-3-*O*-sulphate) equivalents. Sinapic acid-4’-*O*-sulphate and sinapic acid-4’-*O*-glucuronide were quantified as ferulic acid-4’-*O*-sulphate and isoferulic acid-4’-*O*-glucuronide equivalents, respectively. The remaining metabolites were quantified using the reference standard compound or a structurally-related reference compound as previously reported by Calani and colleagues [28].

### Compositional analysis of fecal and cecal microbiota with the HuGChip

Total DNA was extracted from fecal and cecal samples (n = 5 per group) using Qiagen’s DNA Stool Kit (Qiagen, West Sussex, UK). DNA quantifications were performed using a NanoDrop ND-1000 spectrophotometer (NanoDrop Technologies, Wilmington, Germany). Microbial community analysis was performed using the Human Gut Chip (HuGChip) (GSE44752) as described [29]. The HuGChip is a phylogenetic microarray consisting of 4,441 probes targeting 66 bacterial families. In short, prokaryotic 16S rRNA genes were amplified, labeled with Cy3 and Cy5 dyes and hybridized to the array. After washing and scanning, data were extracted and background noise filtered out using Agilent Feature Extraction software 10.5.1.1 (Gene Expression Omnibus Accession: GSE71726).

### Short-chain fatty acids analysis in cecum and in fecal contents

Following appropriate dilution and ultrasonic extraction, the main SCFAs (Acetic, Butyrate, Isobutyrate, Propionate) were analysed using gas chromatography (HP 6890 series, column HPINNOWAX 30 m×250 μm×0.25 μm, split ratio = 25:1, Agilent Technologies). 2-Ethyl-butyrate was used as an internal standard [30].

### Statistical analysis

The difference between the two groups for each fatty acid or each polyphenol metabolite was analyzed using the unpaired Student’s t-test using Minitab software version 15 (GrimmerSoft, Paris, France). Data on relative abundances of bacterial phyla and families as detected by the HuGChip were analyzed using an ANOVA test followed by a Tukey-Kramer post-hoc test, according to time and diet (GraphPad Prism v4 Software, San Diego CA, USA). Differences were considered significant if p<0.05.

Simpson and Shannon Wiener’s diversity indices were calculated using the Paleontological Statistics (PAST) software [31]. Urinary excretion of phenolic metabolites distributions was tested for normality and compared using the Student’s *t*-test (p<0.05). Statistical analyses were performed with SPSS Version 20.0 (SPSS Inc., Chicago, IL, USA).

## Results

### Macronutrient, fatty acid and polyphenol compositions in refined and whole rye diets

Table 1 shows that the WR diet provided more fiber (+21%) and more polyphenols (+29%) than the RR diet, while the other macronutrients did not differ. Dietary fatty acids in both groups did not differ; in particular there was no difference in amounts of *essential* linoleic (18:2n-6) and alpha-linolenic (18:3n-3) acids.

**Table 1:** Macronutrient, fatty acid and polyphenol composition in refined and whole rye diets.

	Refined rye	Whole rye
Macronutrients (g/100g pellet)		
Protein	14.8	14.4
Fat	2.50	2.70
Available carbohydrates	48.0	44.0
Fiber	13.9	16.9
Cellulose	2.30	2.90
Humidity	14.6	14.4
Ash	3.90	4.30
Fatty acids (% of total fatty acids)		
C16:0	14.4	14.6
C18:0	2.72	2.39
C18:1n-9	19.4	19.2
C18:1n-7	1.28	1.34
C18:2n-6	53.7	53.9
C18:3 n-3	6.30	6.39
Total SFA^1^	17.6	17.5
Total MUFA^2^	21.0	20.8
Total (n-3)	7.30	7.44
Total (n-6)	53.9	54.1
Polyunsaturated/saturated	3.47	3.50
Polyphenols (mg/g pellet)		
*Phenolic acids*		
4’-coumaric acid	0.06	0.09
Caffeic acid	0.01	0.01
*Trans*-ferulic acid	1.04	1.59
Sinapic acid	0.03	0.05
Diferulic acids	5.09	7.40
Triferulic acids	0.46	0.71
*Isoflavones*		
Glycitein	0.04	0.05
Daidzein	0.08	0.07
Genistein	0.18	0.15
Daidzein-7-*O*-glucoside	0.13	0.11
Genistein-7-*O*-glucoside	0.37	0.33
Glycitein-7-*O*-glucoside	0.02	nd
*Lignans*		
Conidendrin	0.26	0.43
Trachelogenin	0.09	0.07

^1^: Saturated fatty acids

^2^: Monounsaturated fatty acids; nd: not detected.

Several phenolic acids have been identified based on mass spectral characteristics reported by Calani and colleagues for ferulates and phenolic acids [28] and in Additional Table 1 for lignans and isoflavones. The sum of phenolic acids was 6.7 mg/g for RR and 9.8 mg/g for WR, mostly being ferulate derivatives in bound form (Table 1). Ferulates were by far the most abundant compounds in both WR and RR diets, while other polyphenolic compounds such as isoflavones and lignans were recovered in minor quantities.

### Body weight and food consumption

Mean body weight after 12 weeks (396±31g vs. 397±36g) and average food intake after 12 weeks (26.5±2.6g/day vs. 26.9±3.0g/day) did not change in the RR and WR groups respectively.

### Urinary excretion of polyphenol metabolites

Several urinary polyphenol metabolites have been identified by mass spectral characteristics. However, new urinary metabolites were identified through MS^n^ analysis and reported in additional Table 2, including some isoflavone metabolites resulting from the intake of their parent compounds. Among the many polyphenol metabolites identified in the urine of rats fed either the RR or the WR diet, there was (as expected) a more than 4-fold increase in enterolactone glucuronide in the WR rats (Table 2). The excretion of many metabolites of phenolic acid, and particularly ferulic acid-*O*-sulphate and ferulic acid-*O*-glucuronide, 3-(phenyl)propionic acid-*O*-sulphate and 3-(hydroxyphenyl)propionic acid-*O*-sulphate, caffeic acid and coumaric acid *O*-sulphates and dihydroxyphenylacetic acid, was also higher in the WR rats compared with the RR rats. All data are freely available (http://dx.doi.org/10.6084/m9.figshare.1528159).

**Table 2:** 24-hour urinary phenolic metabolites in refined and whole rye diets.

Compound, μmol excreted	Refined rye	Whole rye
3’-Coumaric acid	**0.03 ±0.01**	0.06 ±0.01^*^
Hydroxyphenylpropionic acid-like	0.18 ±0.04	0.28 ±0.06
3-(3’-Hydroxyphenyl)propionic acid	0.56 ±0.17	1.28 ±0.36
3’,4’-Dihydroxyphenylacetic acid	nd	0.04 ±0.01
Dihydroxyphenylacetic acid	**0.14 ±0.05**	0.39 ±0.07^*^
Hippuric acid	21.90 ±3.33	28.61 ±5.12
3-(3’,5’-Dihydroxyphenyl)propionic acid	nd	0.17 ±0.08
Enterolactone	0.07 ±0.02	0.10 ±0.05
Benzoic acid-*O*-sulphate	0.05 ±0.01	0.06 ±0.01
Coumaric acid-*O*-sulphate	**0.22 ±0.06**	0.57 ±0.13^*^
3-(Phenyl)propionic acid-*O*-sulphate	**0.70 ±0.18**	1.62 ±0.33^*^
Vanillic acid-4-*O*-sulphate	0.06 ±0.02	0.11 ±0.03
Caffeic acid-*O*-sulphate	**0.05 ±0.01**	0.20 ±0.05^*^
3-(Hydroxyphenyl)propionic acid-*O*-sulphate	**0.54 ±0.12**	1.45 ±0.17^*^
Ferulic acid-4’-*O*-sulphate	**0.88 ±0.33**	1.89 ±0.46*
Dihydroferulic acid-4’-*O*-sulphate	**0.03 ±0.01**	0.08 ±0.01^*^
Sinapic acid-4’-*O*-sulphate	0.02 ±0.01	0.04 ±0.01
Ferulic acid-4’-*O*-glucuronide	**0.04 ±0.02**	0.14 ±0.04^*^
Sinapic acid-4’-*O*-glucuronide	0.22 ±0.16	0.66 ±0.21
Enterolactone-*O*-glucuronide	**0.07 ±0.02**	0.32 ±0.10^*^

Data are expressed as mean values ± SEM, n = 6 per group; nd: not detected.

*: indicates p< 0.05.

### Blood lipids, plasma and liver fatty acids profiles

Cholesterol– 0.50±0.08 and 0.53±0.08 g/L in the RR and WR group respectively–and triglycerides– 1.13±0.45 vs. 1.00±0.43 g/L–did not differ in the two groups.

As regards hepatic fatty acid composition, the two major n-3 LCFAs, EPA and DHA, were significantly higher (p<0.05) in the WR rats (Table 3). In addition, there was a significant increase in the level of 18:4n-3 (p<0.05) associated with a nonsignificant decrease in the level of 18:3n-3, these changes suggesting stimulation of the whole n-3 pathway in the liver of WR rats. At the same time, there was a significant increase in the level of 18:3n-6 (p<0.001) and a significant decrease in that of 18:2n-6 (p<0.05) with no other change for other n-6 fatty acids (in particular 20:4n-6 and 22:4n-6); this data suggests partial stimulation of the n-6 pathway in the liver of the WR rats compared with that of the RR rats.

**Table 3:** Plasma lipids and plasma and liver fatty acids (as % of total fatty acids) after 12 weeks.

	Refined rye		Whole rye			Refined rye		Whole rye		
	Plasma					Liver				
Fatty acids (%)										
C14:0	**0.41**	±0.08	**0.22**	±0.08	***	0.18	±0.02	0.22	±0.06	
C16:0	**20.7**	±1.4	**18.7**	±1.8	**	19.5	±0.77	19.2	±0.68	
C16:1n-7	2.53	±0.62	1.95	±0.76		1.38	±0.21	1.54	±0.46	
C18:0	6.95	±0.77	7.66	±1.19		14.7	±0.27	15.0	±1.2	
C18:1n-9	12.3	±2.5	10.6	±2.9		7.68	±0.60	8.34	±1.1	
C18:1n-7	2.56	±0.42	2.33	±0.47		3.47	±0.32	3.29	±0.68	
C18:2n-6	23.7	±1.8	23.1	±1.9		**20.2**	±1.10	**18.8**	±0.73	*
C18:3n-6	0.31	±0.07	0.32	±0.12		**0.19**	±0.02	**0.26**	±0.03	***
C20:0	0.05	±0.02	0.07	±0.05		0.03	±0.005	0.03	±0.003	
C18:3n-3 (α-Linolenic)	1.09	±0.25	1.08	±0.18		0.61	±0.05	0.59	±0.09	
C18:4n-3	0.07	±0.02	0.07	±0.03		**0.03**	±0.01	**0.05**	±0.01	*
C20:2n-6	0.11	±0.02	0.12	±0.02		0.17	±0.02	0.16	±0.02	
C20:3n-9	0.17	±0.05	0.17	±0.03		0.14	±0.04	0.14	±0.03	
C20:3n-6	0.^33^	±0.07	0.36	±0.08		0.49	±0.10	0.51	±0.16	
C20:4n-6	22.6	±5.0	22.6	±5.5		23.5	±0.46	23.3	±1.68	
C20:5n-3 (EPA)	**1.31**	±0.21	**1.51**	±0.23	*	**0.64**	±0.10	**0.75**	±0.06	*
C22:4n-6	0.32	±0.07	0.35	±0.05		0.30	±0.03	0.33	±0.02	
C22:5n-3	0.69	±0.11	0.77	±0.13		0.86	±0.08	0.92	±0.08	
C22:6n-3 (DHA)	**3.84**	±0.47	**4.31**	±0.60	*	**5.75**	±0.32	**6.34**	±0.36	*
Total	**100**		**100**			**100**		**100**		

Values are means ± SD, n = 12 per group for the plasma and n = 6 per group for the liver

*: p<0.05

**: p<0.01

***: p<0.001.

In the plasma, levels of EPA and DHA were also significantly higher (p<0.05) in the WR rats (Table 3). In contrast, compared with the hepatic data, there were no other differences in n-3 or n-6 in the plasma of the WR and RR rats.

Unexpectedly, the levels of two saturated fatty acids (14:0 and 16:0) were significantly lower in the plasma of the WR rats than in that of the RR rats.

### Microbiota profiling of feces and cecum

**Fig 1:**
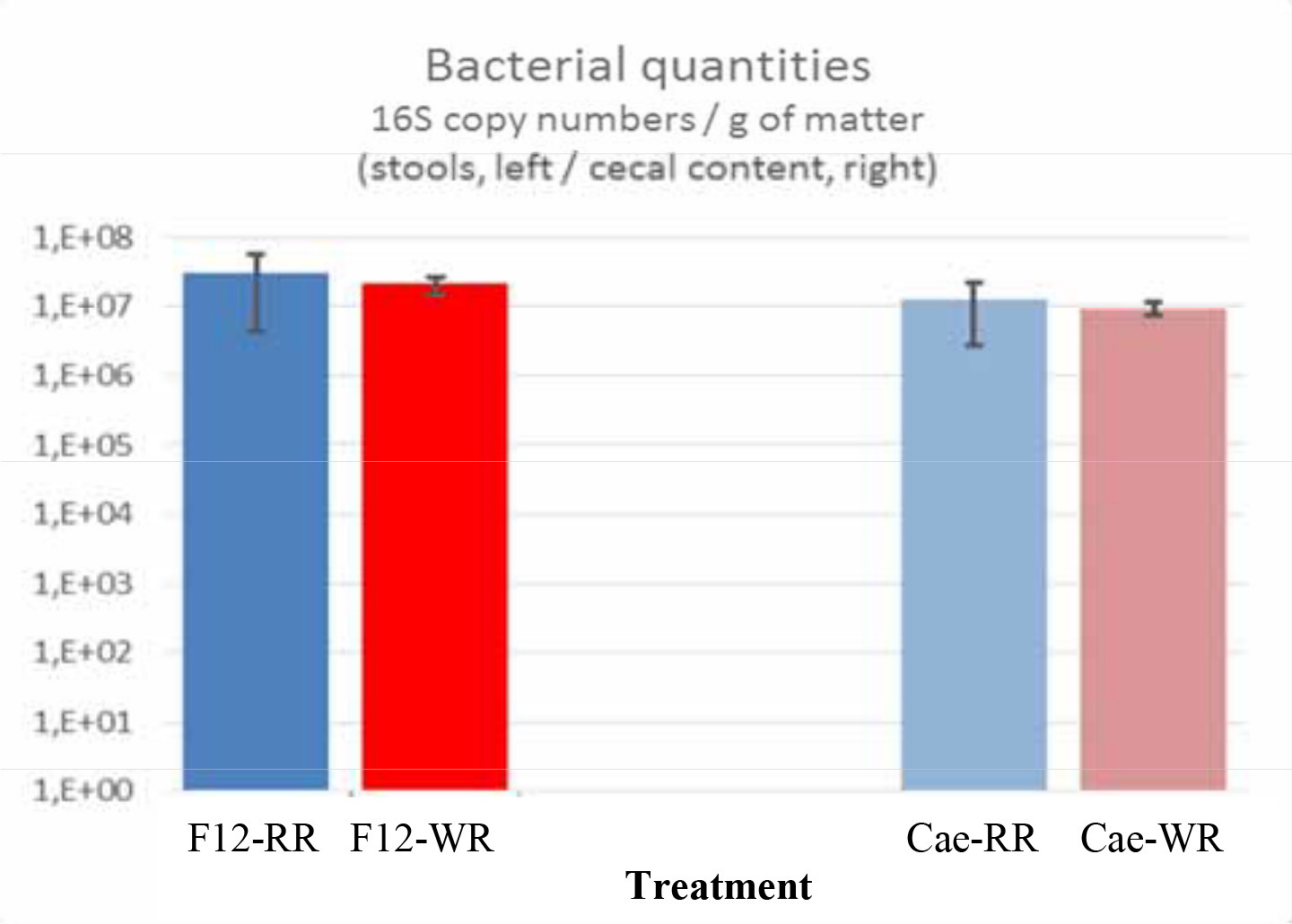
Bacterial quantities in the feces (F12) and in the cecum (Cae) of rats fed a diet containing whole rye (WR) or refined rye (RR).

Fig 1 shows similar bacterial quantities in the feces and in the cecum of rats fed a diet containing WR or RR. Analyses of gut microbiota focused on identifying bacterial phyla and families significantly differing in relative abundance between the two groups at baseline and after 12 weeks, or the abundance of which changed significantly within either the WR or the RR group during the 12-week follow-up (Table 4). The fecal microbiota composition of the two groups was the same at baseline and the factor time alone (0–12 weeks) had no significant effect on the fecal microbiota. Considering the overall diversity of fecal samples, the RR and WR diets induced an opposite effect with a significant increase of the Shannon and Simpson indexes under WR, and a decrease under RR (Table 4).

**Table 4:** Microbial diversity in the feces at the start (F0) and after 12 weeks (F12) in the cecum of rats fed refined or whole rye.

	Refined rye			Whole rye		
	F0	F12^†^	Cecum^*^	F0	F12^†^	Cecum^*^
Simpson index	0.92 ± 0.02	0.89 ± 0.03	0.91 ± 0.02	0.91 ± 0.01	0.94 ± 0.01	0.87 ± 0.05
Shannon index	2.58 ± 0.19	2.25 ± 0.20	2.50 ± 0.25	2.44 ± 0.10	2.88 ± 0.14	2.16 ± 0.37

Values are means ± SD; n = 5 per group. Different from F0

^†^significantly different (P<0.05; ANOVA test with Tukey-Kramer *post-hoc* test) considering (time*diet).

^*^No significant differences observed for the cecal microbiota considering rats fed refined or whole rye.

At the phylum level, no significant effect of time*diet was detected, although a trend was observed for *Firmicutes* (p = 0,067) and *Bacteroidetes* (p = 0,085), both affected by the diets in an opposite manner: an increase of *Firmicutes* and *Bacteroidetes* was observed after 12 weeks of RR and WR diet respectively, and, on the contrary, a decrease with the other diet (Table 5) Some evolutions (cross-effect time*diet) were detected at the family level, encompassing significant increase with the RR diet and a decrease with the WR diet for *Desulfovibrionaceae* and for some *Firmicutes* (*Clostridium* cluster XIV and uncultured *Clostridiales* IA, trend for *Clostridium* cluster IV (p = 0.060)). Inversely, *Neisseriaceae* were decreased under RR diet and increased under WR diet. At the end of the experiment, the cecum content shows no significant differences in diversity and bacterial composition between the two diets. All data are freely available (http://dx.doi.org/10.6084/m9.figshare.1528160).

**Table 5:** Selected bacterial families in the feces at the start (F0) and after 12 weeks (F12) in the cecum (after 12 weeks) of rats fed a diet containing refined rye or whole rye.

Phylum	Family	Refined rye (%)			Whole rye (%)		
		F0	F12	Cecum	F0	F12	Cecum
Firmicutes	*Clostridium* cluster IV	10.5 ± 5.9	15. 4 ± 5.3	12.6 ± 3.7	11.7 ± 2.0	9.6 ± 2.3	13.6 ± 10.4
	*Clostridium* cluster XIV^†^	8.6 ± 1.6	11. 1 ± 4.4	6.5 ± 4.2	9.0 ± 1.4	5.9 ± 1.1	9.5 ± 7.1
	Uncultured *clostridiales* IA^†^	6.7 ± 4.6	9.94 ± 2.0	9.1 ± 2.8	8.9 ± 1.2	6.7 ± 1.5	8.0 ± 4.7
Bacteroidetes	*Bacteroidaceae*	2.0 ± 2. 7	1.5 ± 3.3	3.4 ± 3.2	2.4 ± 3.3	5.1 ± 1.0	1.4 ± 3.1
	*Porphyromonadaceae*	6.9 ± 1.9	6.8 ± 4.3	5.2 ± 3.7	7.3 ± 1.8	6.0 ± 1.8	4.1 ± 4.1
	*Prevotellaceae*	10.1 ± 4.7	9.0 ± 6.5	7.0 ± 7.9	10. 7 ± 2.8	7.0 ± 3.9	14.3 ± 2.9
	*Coriobacteriaceae*	8.8 ± 3.7	9.5 ± 2.9	7.6 ± 4.3	10.4 ± 2.6	7.0 ± 1.5	10.9 ± 8.0
Proteobacteria	*Desulfovibrionaceae*^†^	8.7 ± 4.9	15.0 ± 1.3	9.2 ± 2.2	13.9 ± 1.8	7.5 ± 1.8	11.5 ± 4.1

Values are means ± SD; n = 5 per group.

^†^significantly different in feces (P<0.05; ANOVA test with Tukey-Kramer *post-hoc* test) considering (time*diet). No significant differences observed for the cecal microbiota considering rats fed refined or whole rye.

### Short-chain fatty acids analysis

**Fig 2:**
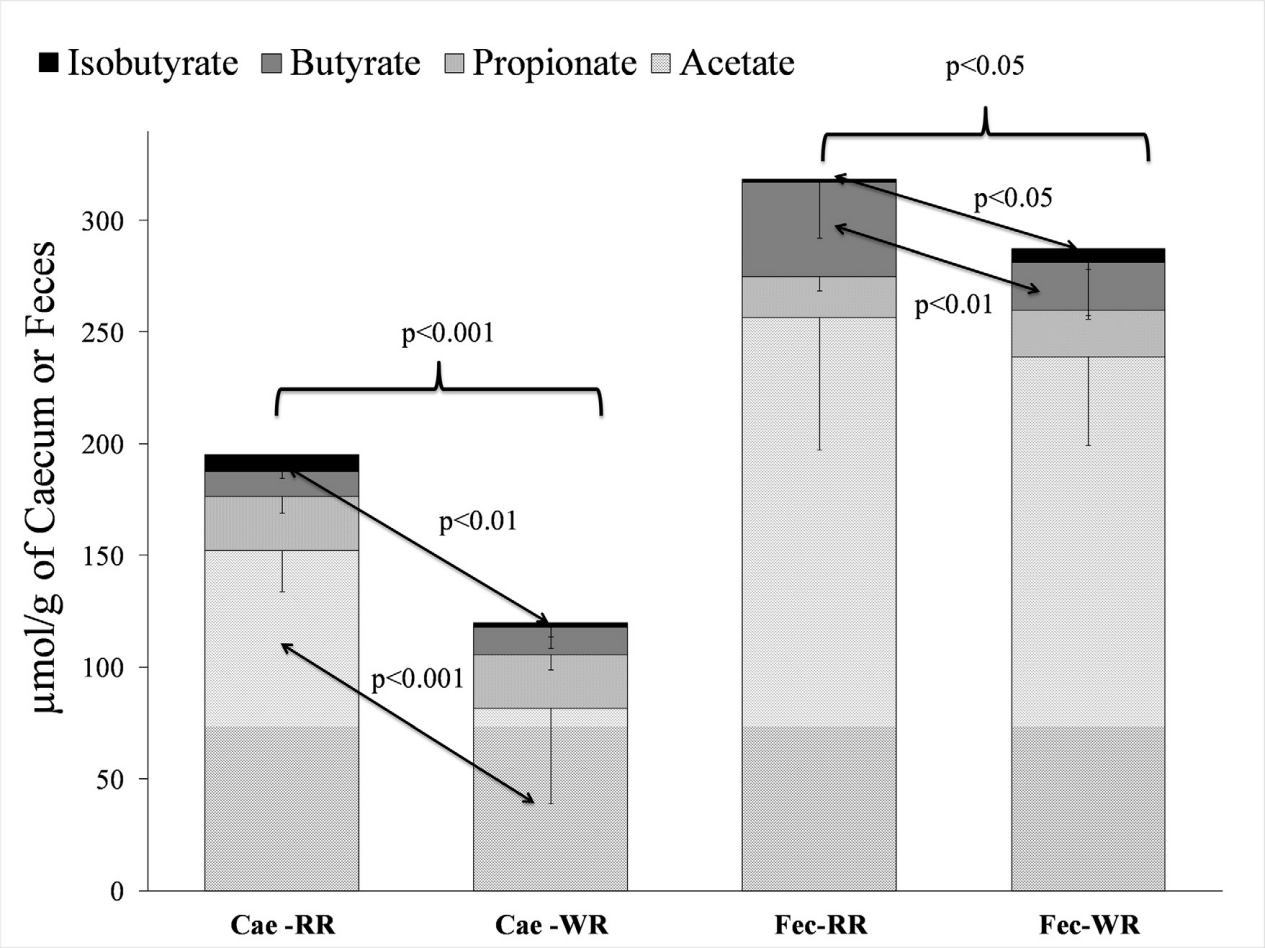
Levels of short chain fatty acids in cecal (Cae) and fecal (Fec) contents after 12 weeks of treatment.

Fig 2 summarizes the main SCFA concentrations in the cecum and feces after 12 weeks. Total SCFA levels were lower in the feces (287 vs. 318 μmol/g, p<0.05) and cecal content (119 vs. 195 μmol/g, p<0.001) of WR rats compared with RR rats. With regard to the cecal SCFA profile, acetate levels were lower in WR rats (82 vs. 152 μmol/g, p<0.001) while isobutyrate was lower (1.9 vs 7.9 μmol/g, p<0.01) in WR rats. In the fecal component, however, isobutyrate was higher (5.6 vs. 1.1 μmol/g, p<0.05) while butyrate was lower (21 vs 42 μmol/g, p<0.01) in WR rats, with no other significant difference as regards other SCFAs. Finally, total SCFA concentration was 1.5 (RR rats) to 2.6 (WR rats) times higher in feces than in cecal content.

## Discussion

### Summary

This study in rats definitely provides new data regarding the effects of WR consumption on lipid metabolism and gut microbiota composition. We show for the first time that WR consumption resulted in increased levels of EPA and DHA in both plasma and liver. The gut microbiota profile was improved with increased overall diversity (as shown by the Simpson and Shannon indexes), decreased *Firmicutes/Bacteroidetes* ratio and decreased *Clostridium* cluster XIV and IA families in WR rats. The total amounts of SCFAs were unexpectedly decreased by WR consumption in both the cecum and feces. These major biological effects can, at least in part, explain the health benefits usually associated with whole grain rye consumption and probably result from the increased consumption of polyphenols and fiber present in large quantities (+29% and +21% respectively) in the WR compared with the RR diet.

### Effect of whole rye on polyphenol excretion in urine

As expected [8], the lignan enterolactone was found to be present in significantly higher levels in the urine of rats fed WR as plant lignans are localized in the aleurone and pericarp-testa cell layers [7], giving a much higher concentration of lignans in the WR than in the RR diet. In the same way, there were significantly more phenolic acid metabolites in the urine of rats treated with WR. It is known that rye bran is a rich source of hydroxycinnamates such as ferulic and coumaric acid [32–34]. It is known that the metabolism of polyphenols, in particular rye lignans, occurs mainly in the gut and mostly under the influence of the microbiota [35, 36] as also discussed below.

### Effect of whole rye on EPA and DHA in liver and plasma

Proportions of plasma and liver n-3 EPA and DHA were significantly higher in rats fed the WR diet. Among the compounds provided by WR, polyphenols may play a dominant role in this. This is an explanation supported by our previous findings on the effects of wine polyphenols in humans and polyphenol-rich cereals in rats, where polyphenol intake was associated with increased plasma n-3 LCFA [13, 14, 26, 27]. A study in patients receiving rye bread also shows increased plasma n-3 LCFA [37] and supports the theory that rye polyphenols influence the metabolism of n-3 LCFA.

In this study, increased proportions of EPA and DHA were observed in the liver for the first time, suggesting that the main impact of WR on the n-3 synthesis pathway may be the stimulation of endogenous n-3 LCFA synthesis in the liver. Indeed, there was a significant increase in 18:4n-3 (2^nd^ step in DHA synthesis) in the liver associated with a nonsignificant decrease in the essential fatty acid 18:3n-3 and a nonsignificant increase in 22:5n-3 (4^th^ step). All of these changes suggest stimulation of the whole n-3 fatty acid synthesis pathway in the liver [38]. In other words, the entire hepatic system involved in n-3 synthesis, including elongases (addition of carbons to the carbonyl chain) and delta-5 and delta-6 desaturase (addition of double-bonds) enzymes is stimulated. At the same time, we observe a significant decrease in the essential fatty acid 18:2n-6 associated with a significant increase in 18:3n-6, suggesting that the first stage of the n-6 synthesis pathway (desaturation) in the liver was stimulated by WR consumption. The next stages of the n-6 synthesis pathway (elongation and second desaturation) were apparently not affected as hepatic and plasma levels of the other n-6 LCFA were not different. Thus, while affecting the same enzymatic system, rye polyphenol consumption affected the n-3 and n-6 synthesis pathways differently. We also reported this as a finding in our previous studies with anthocyanins and wheat phenolic acids [13, 14, 26, 27]. A possible explanation for this is that some steps of the n-3 synthesis pathway, and notably DHA synthesis (last step), is partly sex hormone-dependent [10–12, 39–42]. Rye lignans may partly explain our data in terms of n-3 since they do have some estrogen-like activity [43, 44]. In contrast, the effect of wheat polyphenols, mainly phenolic acids with no estrogen-like effect, on the n-3 synthesis pathway was limited as only plasma EPA was increased [14]. The effect of rye-derived enterolactone is quite similar to that of anthocyanin pigments, that have been demonstrated to increase both EPA and DHA [13, 26, 27]. However, while anthocyanins and enterolactone share some mechanisms of action on the n-3 synthesis pathway, this is probably not through their estrogen-like effect (described with enterolactone) since anthocyanins do not have any estrogen-like effect.

Another potential mechanism of action is the possibility that the antioxidant polyphenols may protect n-3 LCFA from oxidative destruction. This is suggested by studies where polyphenols reduced lipid peroxidation [45]. Finally, in a recent study involving growing gilts fed a flax-seed containing diet [46], ileal flow of EPA and DHA exceeded their dietary intakes, indicating net appearance of these fatty acids and suggesting that gut microbiota could stimulate the n-3 synthesis pathway. In our study, however, we show only a marginal effect on cecal microbiota, suggesting that if the gut microbiota is involved, this predominantly occurs in the large intestine and not in the cecum or above the cecum. Another critical question that arises is why the supposedly EPA (and DHA)-boosting mechanisms work on the n-3 and not the n-6 synthesis pathway, although the same enzymatic equipment and also the same antioxidant (and supposedly protective) polyphenols are involved. While future studies need to be conducted to investigate complex interactive mechanisms, this study clearly confirms a disconnection between n-3 and n-6 pathway regulation under the influence of polyphenols, whilst providing no satisfactory explanation.

### Whole rye-induced modification of the gut microbiota and short-chain fatty acid production

The second main objective of this study was to examine whether WR consumption results in significant changes in overall gut microbiota composition and some associated metabolic effects. This was assessed by analyzing gut bacteria at phylum and family level and measuring SCFA in both feces and cecum. This comparison was done according to the results showing no difference between bacterial quantities observed in the feces and in the cecum. We acknowledge that a more specific approach at species level would have constituted a more precise approach, at least qualitatively, but our main objective was to identify significant changes in overall microbiota composition and diversity following WR consumption rather than very specific changes at species level. In fact the WR diet significantly increased microbial diversity in the feces compared with the RR diet. Diversity is important in all ecosystems in order to promote both stability and adaptation. Gut microbiota diversity is becoming a new health indicator whereas loss of diversity within the gut microbiota has been linked with an increased number of pathological conditions [47, 48]. From these data, it can be said that the increased gut microbiota diversity resulting from WR consumption is definitely a potential health effect of WR diet.

After 12 weeks of this study, a significant lower proportion of the phylum *Firmicutes* was found in the WR rats, combined with a higher proportion of the phylum *Bacteroidetes* although non-significant (p = 0.085) and thus a lower *Firmicutes*-to-*Bacteroidetes* ratio, which is often, although not always, considered beneficial for the prevention of metabolic disorders such as obesity and insulin resistance [22, 49–52]. Concomitantly the number of *Firmicutes (*especially *Clostridium* cluster XIV and uncultured *Clostridiales* IA) decreased and the number of *Bacteriodetes* (especially Bacteroidaceae) increased when following the WR diet, whereas the opposite was observed in rats following the RR diet. This is in line with what has been observed during studies in humans [53], indicating that a plant-based diet is associated with decreased *Clostridium* cluster XIV and a higher *Bacteroides*-*Prevotella* ratio. In our study the WR diet resulted in a more than 3-fold increase in mean *Bacteroidaceae*-*Prevotellaceae* ratio (0.72), while this was unaffected by the RR diet: 0.17 compared to 0.20 and 0.22 at baseline. By contrast, an inverse effect is observed in the cecum, this ratio being 0.10 and 0.49 after 12 weeks under the WR and the RR diet respectively (Table 5). This highlights the importance of assessing the cecum in rat experiments, as further indicated by SCFA production: under the WR diet, cecal SCFAs are reduced (compared with RR diet) and also differ qualitatively from the SCFAs retrieved from the feces (Fig 2). These data are difficult to interpret because we cannot separate two different phenomena in the gut (particularly above the cecum), i.e. the production and intestinal absorption of SCFA. More investigation must be done to link the SCFA profile in the gut with the SCFA profile in the body (suggesting potential health benefits). However this could suggest that the microbiota of WR rats may be less efficient at extracting energy from a given diet than the microbiota of RR rats, even in presence of nondigestible carbohydrates (fiber).

Regarding specifically the SCFA issue, the main difference between the two groups observed in our study concerns the absolute amounts of acetate in the cecum and of butyrate in the feces. These results are in line with recent studies investigating the interaction between gut bacteria and polyphenols [54, 55]. In a study in rats, dietary polyphenols affected the growth of certain families of bacteria, including *Bifidobacterium* and *Prevotella*, with a reduction in cecal acetate levels, as in our own study [54]. In another study in humans, anthocyanin-rich wine increased the fecal concentration of *Bifidobacterium* and the highest proportions of *Bifidobacteria* were associated with the highest urinary concentrations of anthocyanin metabolites, confirming these polyphenols as potential bacterial substrates [55]. These studies highlight the importance of identifying and quantifying all polyphenol metabolites in urine and feces using specific techniques such as isotopically labeled cyanidin-3-glucoside in humans [56]. Overall these data indicate that both the nondigestible carbohydrates (fiber) and the polyphenols in the WR diet were potential contributors to the changes observed in gut microbiota composition and SCFA production.

We also note that, in general, our data do not agree with certain studies in humans showing that the fecal microbiotas of humans living in rural areas are enriched with *Prevotella* spp. [57, 58]. The difference may be due to long-term exposure to traditional diets rich in plant-derived complex carbohydrates but poor in total energy intake [57, 58]. Our results contrast with those observed in humans eating whole rye bread, where fecal proportions of the *Bacteroides* species decreased after 6 weeks [59]. However, in this study patients had metabolic syndrome at baseline (and supposedly the associated microbiota) and exposure to whole rye bread was very short-term (6 weeks) and not long enough to provoke significant changes in microbiota composition. These examples highlights that studies should be carefully examined before drawing any conclusions or before extrapolating animal data to humans. Finally, the *Proteobacteria* phylum increased in WR rats after 12 weeks despite a significant drop in the *Desulfovibrionaceae* family. This might be beneficial to health as sulfate-reducing bacteria produce hydrogen sulfide, whose effects in the gut are unclear but thought to be harmful [60, 61]. Viewed as a whole, our data illustrate the complexity of gut microbiota physiology and the way the gut bacteria respond to dietary changes. Further studies using more sophisticated technology are required in order to fully understand how WR influences gut microbiota and how in turn the gut microbiota influences the metabolism of plant polyphenols. In particular, the effect of gut microbiota on SCFA production is complex and cannot be explained by the stimulation of a single phylum such as the *Firmicutes* or specific *Firmicutes* families either above or below the cecum.

Taken all together, our results showed that WR consumption is associated with biological modifications considered as health benefits. Indeed, it increased plasma and liver n-3 EPA and DHA levels and improved gut microbiota profile, especially by increasing their diversity known for their benefits against chronic diseases [9–22, 23].

## Conclusions

Including WR in the diet increased plasma and hepatic concentrations of n-3 LCFA (as well as urinary polyphenols), improved Firmicutes/Bacteroidetes ratio's and gut microbiota diversity. These results demonstrated for the first time that WR consumption can result in several biological modifications with potential health benefits. Although the exact mechanisms are not clearly identified and require further investigation, our results provide new insights into the possible health benefits of whole rye consumption. For instance, the effect of WR consumption on rats with metabolic syndrome can be investigated.

## Supporting Information

S1 Fig Bacterial phyla in the faeces at the beginning (F0) and at the end of the experiment (F12) and in the caecum (Cae) of rats fed a diet containing whole rye (WR) or refined rye (RR). (EPS)

S1 Table Mass spectral characteristics of isoflavones and lignan in rye diets. (EPS)

S2 Table Mass spectral chracteristics of urinary polyphenolic metabolites. (EPS)

## ABBREVIATIONS

RR: refined rye
WR: whole rye
Cae: Cecal content
HuGChip: Human Gut Chip
PAST: Paleontological Statistics
SCFA: short chain fatty acids
EPA: Eicosapentaenoic acid
DHA: Docosahexanoic acid
UHPLC: Ultra High Performance Liquid Chromatography

## References

[1] Wu H, Flint AJ, Qi Q, van Dam RM, Sampson LA, Rimm EB, et al Association between dietary whole grain intake and risk of mortality. JAMA Intern Med. 2015; 175(3):373–384. 10.1001/jamainternmed.2014.6283 25559238 PMC4429593

[2] Bartłomiej S, Justyna RK, Ewa N. Bioactive compounds in cereal grains—occurrence, structure, technological significance and nutritional benefits—a review. Food Sci Technol Int. 2012; 18: 559–568. 10.1177/1082013211433079 23064524

[3] Belobrajdic DP, Bird AR The potential role of phytochemicals in wholegrain cereals for the prevention of type-2 diabetes. Nutr J. 2013; 12:62 10.1186/1475-2891-12-62 23679924 PMC3658901

[4] Frølich W, Aman P, Tetens I. Whole grain foods and health—a Scandinavian perspective. Food Nutr Res. 2013; 57: 18503. 10.3402/fnr.v57i0.18503 PMC3572214 23411562

[5] Kyrø C, Skeie G, Dragsted LO, Christensen J, Overvad K, Hallmans G, et al Intake of whole grain in Scandinavia: intake, sources and compliance with new national recommendations. Scand J Public Health. 2012; 40(1):76–84. 10.1177/1403494811421057 21976053

[6] Glitsø LV, Mazur WM, Adlercreutz H, Wähälä K, Mäkelä T, Sandström B, et al Intestinal metabolism of rye lignans in pigs. Br J Nutr. 2000; 84(4):429–37. 11103213

[7] Nilsson M, Åman P, Härkönen H, Hallmans G, Knudsen KEB, Mazur W, et al Content of nutrients and lignans in roller milled fractions of rye. J Sc of Food and Agri. 1997; 73(2): 143–148.

[8] Juntunen KS, Mazur WM, Liukkonen KH, Uehara M, Poutanen KS, Adlercreutz HC, et al Consumption of whole meal rye bread increases serum concentrations and urinary excretion of enterolactone compared with consumption of white wheat bread in healthy Finnish men and women. Br J Nutr. 2000; 84(6):839–46. 11177200

[9] Superko HR, Superko SM, Nasir K, Agatson A, Garrett BC. Omega-3 fatty acid blood levels. Clinical significance and controversy. Circulation. 2013; 128:2154–61. 10.1161/CIRCULATIONAHA.113.002731 24190935

[10] Childs CE, Romeu-Nadal M, Burdge GC, Calder PC. Gender differences in the n-3 fatty acid content of tissues. Proc Nutr Soc. 2008; 67(1):19–27. 10.1017/S0029665108005983 18234128

[11] Burdge GC, Calder PC. Conversion of alpha-linolenic acid to longer-chain polyunsaturated fatty acids in human adults. Review. Reprod Nutr Dev. 2005; 45(5):581–97. 16188209 10.1051/rnd:2005047

[12] Sibbons CM, Brenna JT, Lawrence P, Hoile SP, Clarke-Harris R, Lillycrop KA, et al Effect of sex hormones on n-3 polyunsaturated fatty acid biosynthesis in HepG2 cells and in human primary hepatocytes. Prostaglandins Leukot Essent Fatty Acids. 2014; 90(2–3):47–54. 10.1016/j.plefa.2013.12.006 24411721 PMC4046896

[13] Toufektsian MC, Salen P, Tonelli C, de Lorgeril M. Dietary flavonoids increase plasma very long-chain (n-3) fatty acids in rats. J Nutr. 2011; 141:37–41. 10.3945/jn.110.127225 21068183

[14] Ounnas F, Privé F, Salen P, Hazane-Puch F, Laporte F, Fontaine E, et al Wheat aleurone polyphenols increase plasma eicosapentaenoic acid in rats. Food Nutr Res. 2014; 58: 24604. 10.3402/fnr.v58.24604 PMC4139929 25206320

[15] Le Gall M, Serena A, Jørgensen H, Theil PK, Bach Knudsen KE**.** The role of whole-wheat grain and wheat and rye ingredients on the digestion and fermentation processes in the gut-a model experiment with pigs. Br J Nutr**.** 2009; 102(11):1590–600. 10.1017/S0007114509990924 19635175

[16] Theil PK, Jørgensen H, Serena A, Hendrickson J, Bach Knudsen KE. Products deriving from microbial fermentation are linked to insulinaemic response in pigs fed breads prepared from whole-wheat grain and wheat and rye ingredients. Br J Nutr. 2011; 105(3):373–83. 10.1017/S0007114510003715 20923581

[17] Lozupone CA, Stombaugh JI, Gordon JI, Jansson JK, Knight R. Diversity, stability and resilience of the human gut microbiota. Nature. 2012; 489(7415):220–30. 10.1038/nature11550 22972295 PMC3577372

[18] Glitsø LV, Brunsgaard G, Højsgaard S, Sandström B, Bach Knudsen KE. Intestinal degradation in pigs of rye dietary fibre with different structural characteristics. Br J Nutr. 1998; 80(5):457–68. 9924268

[19] Wong JM, de Souza R, Kendall CW, Emam A, Jenkins DJ. Colonic health: fermentation and short chain fatty acids. J Clin Gastroenterol. 2006; 40(3):235–43. 16633129 10.1097/00004836-200603000-00015

[20] Hamer HM, Jonkers D, Venema K, Vanhoutvin S, Troost FJ, Brummer RJ, et al Review article: the role of butyrate on colonic function. Aliment Pharmacol Ther. 2008; 15;27(2):104–19. 17973645 10.1111/j.1365-2036.2007.03562.x

[21] Everard A, Cani PD. Diabetes, obesity and gut microbiota. Best Pract Res Clin Gastroenterol. 2013; 27(1):73–83. 10.1016/j.bpg.2013.03.007 23768554

[22] Zhao L. The gut microbiota and obesity: from correlation to causality. Nat Rev Microbiol. 2013; 11(9):639–47. 10.1038/nrmicro3089 23912213

[23] Freeland KR, Wilson C, Wolever TM. Adaptation of colonic fermentation and glucagon-like peptide-1 secretion with increased wheat fibre intake for 1 year in hyperinsulinaemic human subjects. Br J Nutr. 2010; 103(1):82–90. 10.1017/S0007114509991462 19664300

[24] Hallmans G, Zhang JX, Lundin E, Stattin P, Johansson A, Johansson I, et al Rye, lignans and human health. Proc Nutr. 2003; Soc 62: 193–119. 10.1079/pns2002229 12749346

[25] Reeves PG, Nielsen FH, Fahey GC. AIN-93 purified diets for laboratory rodents: final report of the American Institute of 365 Nutrition ad hoc writing committee on the reformulation of the AIN-76A rodent diet. J Nutr. 1993; 123: 193951. 10.1093/jn/123.11.1939 8229312

[26] di Giuseppe R, de Lorgeril M, Salen P, Laporte F, Di Castelnuovo A, Krogh V, et al Alcohol consumption and n-3 polyunsaturated fatty acids in healthy men and women from 3 European populations. Am J Clin Nutr. 2009; 89:354–62. 10.3945/ajcn.2008.26661 19056552

[27] de Lorgeril M, Salen P, Martin JL, Boucher F, de Leiris J. Interactions of wine drinking with omega-3 fatty acids in patients with coronary heart disease: a fish-like effect of moderate wine drinking. Am Heart J. 2008; 155:175–81. 18082510 10.1016/j.ahj.2007.08.009

[28] Calani L, Ounnas F, Salen P, Demeilliers C, Bresciani L, Brighenti F, et al Bioavailability and metabolism of hydroxycinnamates in rats fed with durum wheat aleurone fractions. Food Funct. 2014; 23;5(8):1738–46. 10.1039/c4fo00328d 24977665

[29] Tottey W, Denonfoux J, Jaziri F, Parisot N, Missaoui M, Hill DRC, et al The human gut chip "HuGChip", an explorative phylogenetic microarray for determining gut microbiome diversity at family level. PLoS One. 2013; 17;8(5):e62544 10.1371/journal.pone.0062544 23690942 PMC3656878

[30] Feria-Gervasio D, Denis S, Alric M, Brugère JF. In vitro maintenance of a human proximal colon microbiota using the continuous fermentation system P-ECSIM. Appl Microbiol Biotechnol. 2011; 91(5):1425–33. 10.1007/s00253-011-3462-5 21773764

[31] Hammer Ø, Haprper DAT, Ryan PD. Paleontological statistics software package for education and data analysis. Palaeontologia Electronica. 2001; 4(1): 4–8.

[32] Andreasen MF, Kroon PA, Williamson G, Garcia-Conesa MT. Esterase activity able to hydrolyze dietary antioxidant hydroxycinnamates is distributed along the intestine of mammals. J Agric Food Chem. 2001; 49(11):5679–84. 11714377 10.1021/jf010668c

[33] Andreasen MF, Christensen LP, Meyer AS, Hansen A. Content of phenolic acids and ferulic acid dehydrodimers in 17 rye (Secale cereale L.) varieties. J Agric Food Chem. 2000; 48(7):2837–42. 11032481 10.1021/jf991266w

[34] Nordlund E, Aura AM, Mattila I, Kössö T, Rouau X, Poutanen K, et al Formation of phenolic microbial metabolites and short-chain fatty acids from rye, wheat, and oat bran and their fractions in the metabolical in vitro colon model. J Agric Food Chem. 2012; 22;60(33):8134–45. 10.1021/jf3008037 22731123

[35] Parkar SG, Trower TM, Stevenson DE. Fecal microbial metabolism of polyphenols and its effects on human gut microbiota. Anaerobe. 2013; 23:12–9. 10.1016/j.anaerobe.2013.07.009 23916722

[36] Landete JM. Plant and mammalian lignans: a review of source, intake, metabolism, intestinal bacteria and health. Food Res Int. 2012; 46:410–424

[37] Lankinen M, Schwab U, Gopalacharyulu PV, Seppänen-Laakso T, Yetukuri L, Sysi-Aho M, et al Dietary carbohydrate modification alters serum metabolic profiles in individuals with the metabolic syndrome. Nutr Metab Cardiovasc Dis. 2010; 20(4):249–57. 10.1016/j.numecd.2009.04.009 19553094

[38] Emken EA. Stable isotope approaches, applications and issues related to polyunsaturated fatty acid metabolism studies. Lipids. 2001; 36: 965–973. 11724469 10.1007/s11745-001-0807-4

[39] Giltay EJ, Gooren LJ, Toorians AW, Katan MB, Zock PL. Docosahexaenoic acid concentrations are higher in women than in men because of estrogenic effects. Am J Clin Nutr. 2004; 80:1167–74. 15531662 10.1093/ajcn/80.5.1167

[40] Burdge GC, Wootton SA Conversion of alpha-linolenic acid to eicosapentaenoic, docosapentaenoic and docosahexaenoic acids in young women. Br J Nutr. 2002; 88(4):411–20. 12323090 10.1079/BJN2002689

[41] Burdge GC, Jones AE, Wootton SA Eicosapentaenoic and docosapentaenoic acids are the principal products of alpha-linolenic acid metabolism in young men. Br J Nutr. 2002; 88(4):355–63. 12323085 10.1079/BJN2002662

[42] Pawlosky R, Hibbeln J, Lin Y, Salem N. n-3 fatty acid metabolism in women. Br J Nutr. 2003; 90(5):993–4; 994–5 14667193 10.1079/bjn2003985

[43] Penttinen P, Jaehrling J, Damdimopoulos AE, Inzunza J, Lemmen JG, van der Saag P, et al Diet derived polyphenol metabolite enterolactone is a tissue-specific estrogen receptor activator. Endocrinology. 2007; 148:4875–86. 17628008 10.1210/en.2007-0289

[44] Damdimopoulou P, Nurmi T, Salminen A, Damdimopoulos AE, Kotka M, van der Saag P, et al A single dose of enterolactone activates estrogen signaling and regulates expression of circadian clock genes in mice. J Nutr. 2011; 141(9):1583–9. 10.3945/jn.111.140277 21753063

[45] Gobert M, Martin B, Ferlay A, Chilliard Y, Graulet B, Pradel P, et al Plant polyphenols associated with vitamin E can reduce plasma lipoperoxidation in dairy cows given n-3 polyunsaturated fatty acids. J Dairy Sci. 2009; 92(12):6095–104. 10.3168/jds.2009-2087 19923612

[46] Martínez-Ramírez HR, Kramer JK, de Lange CF. Ileal flows and apparent ileal digestibility of fatty acids in growing gilts fed flaxseed containing diets. J Anim Sci. 2013; 91(6):2729–39. 10.2527/jas.2012-5783 23478834

[47] Le Chatelier E, Nielsen T, Qin J, Prifti E, Hildebrand F, Falony G, et al Richness of human gut microbiome correlates with metabolic markers. Nature. 2013; 500:541–546. 10.1038/nature12506 23985870

[48] Claesson MJ, Jeffery IB, Conde S, Power SE, O'Connor EM, Cusack S, et al Gut microbiota composition correlates with diet and health in the elderly. Nature. 2012; 488:178–184. 10.1038/nature11319 22797518

[49] Bervoets L, Van Hoorenbeeck K, Kortleven I, Van Noten C, Hens N, Vael C, et al Differences in gut microbiota composition between obese and lean children: a cross-sectional study. Gut Pathog. 2013; 5(1):10 10.1186/1757-4749-5-10 23631345 PMC3658928

[50] Turnbaugh PJ, Ley RE, Mahowald MA, Magrini V, Mardis ER, Gordon JI. An obesity-associated gut microbiome with increased capacity for energy harvest. Nature. 2006; 444(7122):1027–31. 17183312 10.1038/nature05414

[51] Turnbaugh PJ, Backhed F, Fulton L, Gordon JI. Diet-induced obesity is linked to marked but reversible alterations in the mouse distal gut microbiome. Cell Host Microbe. 2008; 3:213–23. 10.1016/j.chom.2008.02.015 18407065 PMC3687783

[52] Caricilli AM, Saad MJ. The role of gut microbiota on insulin resistance. Nutrients. 2013; 5(3):829–51. 10.3390/nu5030829 23482058 PMC3705322

[53] Matijašić BB, Obermajer T, Lipoglavšek L, Grabnar I, Avguštin G, Rogelj I. Association of dietary type with fecal microbiota in vegetarians and omnivores in Slovenia. Eur J Nutr. 2014; 53(4):1051–64. 10.1007/s00394-013-0607-6 24173964

[54] Unno T, Sakuma M, Mitsuhashi S. Effect of dietary supplementation of (-)-epigallocatechin gallate on gut microbiota and biomarkers of colonic fermentation in rats. J Nutr Sci Vitaminol (Tokyo). 2014; 60(3):213–9. 25078378 10.3177/jnsv.60.213

[55] Boto-Ordóñez M, Urpi-Sarda M, Queipo-Ortuño MI, Tulipani S, Tinahones FJ, Andres-Lacueva C. High levels of Bifidobacteria are associated with increased levels of anthocyanin microbial metabolites: a randomized clinical trial. Food Funct. 2014; 5:1932–1938. 10.1039/c4fo00029c 24958563

[56] Czank C, Cassidy A, Zhang Q, Morrison DJ, Preston T, Kroon PA, et al Human metabolism and elimination of the anthocyanin, cyanidin-3-glucoside: a (13)C-tracer study. Am J Clin Nutr. 2013; 97:995–1003. 10.3945/ajcn.112.049247 23604435

[57] De Filippo C, Cavalieri D, Di Paola M, Ramazzotti M, Poullet JB, Massart S, et al Impact of diet in shaping gut microbiota revealed by a comparative study in children from Europe and rural Africa. Proc Natl Acad Sci U S A. 2010; 107(33):14691–6. 10.1073/pnas.1005963107 20679230 PMC2930426

[58] Yatsunenko T, Rey FE, Manary MJ, Trehan I, Dominguez-Bello MG, Contreras M, et al Human gut microbiome viewed across age and geography. Nature. 2012; 486(7402):222–7. 10.1038/nature11053 22699611 PMC3376388

[59] Lappi J, Salojärvi J, Kolehmainen M, Mykkänen H, Poutanen K, de Vos WM, et al Intake of whole-grain and fiber-rich rye bread versus refined wheat bread does not differentiate intestinal microbiota composition in Finnish adults with metabolic syndrome. J Nutr. 2013; 143(5):648–55. 10.3945/jn.112.172668 23514765

[60] Carbonero F, Benefiel AC, Alizadeh-Ghamsari AH, Gaskins HR. Microbial pathways in colonic sulfur metabolism and links with health and disease. Front Physiol. 2012; 3:448 10.3389/fphys.2012.00448 23226130 PMC3508456

[61] Rey FE, Gonzalez MD, Cheng J, Wu M, Ahern PP, Gordon JI, et al Metabolic niche of a prominent sulfate-reducing human gut bacterium. Proc Natl Acad Sci U S A. 2013; 110(33):13582–7. 10.1073/pnas.1312524110 23898195 PMC3746858

